# Inflammatory Arthritis Associated with COVID-19 Vaccination

**DOI:** 10.7759/cureus.35951

**Published:** 2023-03-09

**Authors:** Sai Man Mung, Tee Lin Goh, Michael Hughes, Edward B Jude

**Affiliations:** 1 Diabetes and Endocrinology, Royal Preston Hospital, Preston, GBR; 2 Endocrinology, Tameside and Glossop Integrated Care NHS Foundation Trust, Ashton under Lyne, GBR; 3 Musculoskeletal and Dermatological Sciences, University of Manchester, Manchester, GBR; 4 Endocrinology, University of Manchester, Manchester, GBR

**Keywords:** vaccine adverse effect, astrazeneca covid vaccine, vaccination related arthritis, covid-19, inflammatory arthritis

## Abstract

COVID-19 vaccines have been shown to be highly efficacious in preventing symptomatic COVID-19 infections throughout the pandemic. There have been emerging cases of inflammatory arthritis occurring in close relation to COVID-19 vaccination. We illustrate a case of new-onset inflammatory arthritis 10 days after receiving their second Vaxzevria COVID-19 vaccine. The patient responded dramatically to prednisolone treatment but subsequently required hydroxychloroquine due to persistent inflammatory joint symptoms. Inflammatory arthritis is an increasingly recognized rare adverse effect of COVID-19 vaccination and clinicians should actively consider this in patients with new or flares of inflammatory joint disease.

## Introduction

COVID-19 is an infectious disease that first emerged in Wuhan, China, in December 2019 and caused by Severe Acute Respiratory Syndrome Coronavirus-2 (SARS-CoV-2) [1]. Due to the rapid spread of this infectious disease and its catastrophic effect on the world’s population, the WHO declared COVID-19 a pandemic and a global health emergency [2]. The first COVID-19 case was reported in the United Kingdom in January 2020 and it is still an going global pandemic [3]. The COVID-19 vaccination was introduced to the world to provide immunity against SARS-CoV-2.

One of the COVID-19 vaccines approved by the Medicines and Healthcare Products Regulatory Agency (MHRA) in the United Kingdom is Vaxzevria, which works by delivering the genetic code of the SARS-CoV-2 spike protein to the body’s cells [4]. A two-dose regime of COVID-19 vaccine has been reported to have efficacy of up to 95% in preventing symptomatic COVID-19 among adults who are aged 16 years and above [4,5]. A wide range of common complications associated with COVID-19 vaccination, including myalgia, headache, and rarely post-vaccination thrombotic events, have been reported [4]. A number of rheumatological conditions including inflammatory arthritis have been reported in the extant literature with close reported association with COVID-19 vaccination. The case presented here reports a patient with significant persistent inflammatory arthritis post-vaccination, who was also found to be weakly anti-proteinase 3 antineutrophil cytoplasmic antibody (ANCA-PR3) positive, without evidence of extra-articular systemic vasculitis.

## Case presentation

A 71-year-old Caucasian man presented to hospital four weeks after new onset of polyarthritis and stiffness lasting for hours initially affecting the small joints of his hands and eventually his feet. He denied any preceding infective illness or previous episodes of inflammatory arthritis. However, he reported that he had received his second dose of the Vaxzevria COVID-19 vaccination 10 days prior to the onset of his symptoms. The close temporal relationship between the development of his inflammatory arthritis and his COVID-19 vaccination could not be excluded. There were no reported adverse effects, including inflammatory symptoms, following his first COVID-19 vaccination.

His past medical history included treated hypertension and type 2 diabetes mellitus. He was an ex-smoker with 20 pack years. His father was previously diagnosed with rheumatoid arthritis; however, the patient himself was not known to have previous rheumatoid arthritis or any autoimmune disease. He denied any recent new medications or recent foreign travel. Systemic review was otherwise unremarkable including conditions related to spondyloarthritis. There were no features to suggest an autoimmune connective tissue disease including episodes of Raynaud’s phenomenon or systemic vasculitis.

**Figure 1:**
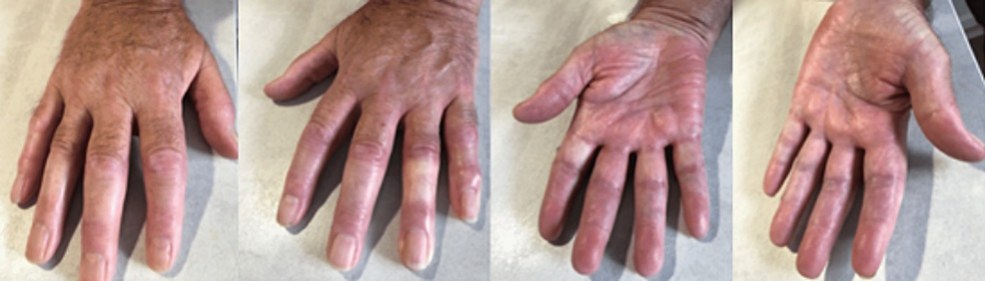
Synovitis of the wrist, proximal interphalangeal joints (PIPJs) and metacarpophalangeal joints (MCPJs) was present on both hands.

On clinical examination, there was florid synovitis of the wrists, proximal interphalangeal joints (PIPJs) and metacarpophalangeal joints (MCPJs) of both hands (Figure 1).

There were no signs of joint inflammation involving his large joints. There was no visible nailfold capillaroscopic abnormality and no evidence of skin thickening such as sclerodactyly. There was no evidence of inflammatory eye disease and no vasculitic rashes. Cardiovascular, respiratory, intestinal and neurological examinations were grossly unremarkable.

His laboratory investigations (Table 1) showed elevated neutrophilia with C-reactive protein (CRP) of 62 mg/L and erythrocyte sedimentation rate (ESR) of 26 mm/hour. His ANCA-PR3 level was mildly raised at 7.4 AI (normal: 0.0-0.9). Rheumatoid factor and anti-cyclic citrullinated peptide (anti-CCP) antibodies were both negative. Serum creatine kinase (CK) and uric acid were normal. Biochemistry including renal function was normal. Viral screen was negative for hepatitis B and C, Epstein-Barr virus (EBV), Cytomegalovirus (CMV) and COVID-19 polymerase chain reaction (PCR).

**Table 1:** Results of laboratory investigations ANCA-MPO: Perinuclear anti-neutrophil cytoplasmic antibodies; ANCA-PR3: Anti-proteinase 3 antineutrophil cytoplasmic antibody; CCP: Cyclic citrullinated peptide; CK: Creatine kinase; CRP: C-reactive protein; eGFR: Estimated glomerular filtration rate; ENA: Extractable nuclear antigen; ESR: Erythrocyte sedimentation rate; RNP: SM: Anti-Smith antibody

Lab investigation	Patient result	Reference range
Full Blood Count		
Haemoglobin	146 g/L	130-180 g/L
White Cell Count	13.5 x10^9^/L	3.6 - 11.0 x 10^9^/L
Neutrophil Count	10.6 x 10^9^/L	1.8 - 7.5 x 10^9^/L
Platelet Count	199 x 10^9^/L	140 - 400 x 10^9^/L
Urea and Electrolytes		
Creatinine	55 umol/L	
eGFR	>60 ml/min	
CRP	62 mg/L	0-8 mg/L
ESR	41 mm/h	< 30 mm/h
Plasma Urate Level	228 μmol/L	200-430 μmol/L
CK	62 U/L	40-320 U/L
Immunology		
ANCA-MPO	<0.2 (AI)	<0.9 (AI)
ANCA-PR3	7.4 (AI)	<0.9 (AI)
Extractable Nuclear Antigens		
Antibody to Chromatin Level	<0.2 (AI)	<0.9 (AI)
Antibody to SM Level	<0.2 (AI)	<0.9 (AI)
Antibody to SM/RNP Level	<0.2 (AI)	<0.9 (AI)
Antibody to SS-A (Ro) Level	<0.2 (AI)	<0.9 (AI)
Antibody to SS-A52 Level	<0.2 (AI)	<0.9 (AI)
Antibody to SS-A60 Level	<0.2 (AI)	<0.9 (AI)
Antibody to SS-B (La) Level	<0.2 (AI)	<0.9 (AI)
ENA scl-70 Ab Level, Serum	<0.2 (AI)	<0.9 (AI)
Jo-1 Ab Level, Serum	<0.2 (AI)	<0.9 (AI)
Ribosomal Auto Ab-Screen	<0.2 (AI)	<0.9
U1-snRNP Ab Level, Serum	<0.2 (AI)	<0.9
Anti-nuclear Antibody	Negative	
Centromere Antibody	<0.2 (AI)	0.0-0.9 (AI)
Rheumatoid Factor Level	<10 (IU/mL)	< 20 (IU/mL)
Anti-CCP	<7 U/mL	<7 U/mL

Chest radiograph was normal. Urinalysis was unremarkable including no proteinuria or haematuria. Radiographs of both hands at baseline did not demonstrate any bony erosions.

Against this background, the clinical impression was of a symmetrical undifferentiated inflammatory polyarthritis which occurred in close reported proximity of his second Vaxzevria COVID-19 vaccination. He had evidence of a systemic inflammatory response with elevated inflammatory markers. Rheumatoid and connective tissue disease serology was negative. Of potential mechanistic interest, he was found to be persistently weakly positive for ANCA-PR3, and with no extra-articular systemic features suggestive of ANCA-associated vasculitis. He was commenced on oral steroid (prednisolone) therapy: initially 30 mg daily on a reducing regimen, along with appropriate gastric and bone protection, and close observation of his diabetic control.

At initial outpatient follow-up consultation, he stated that he felt prompt 95% improvement of his symptoms after the commence of prednisolone. CRP promptly normalised and the ANCA-PR3 level remains at low-titre (approximately 4.5 AI) without new systemic manifestations of ANCA-associated vasculitis. He was commenced on hydroxychloroquine due to persistent low-grade inflammatory joint disease and remains on low-dose (currently 5 mg daily) prednisolone, with a view to discontinuation in the near future.

## Discussion

COVID-19 vaccination is vital in combating this highly contagious viral illness and controlling the COVID-19 pandemic. However, COVID-19 vaccinations can be associated with complications in a minority of the population, such as local, systemic or autoimmune complications. [6].

Vaccination-related new onset autoimmune joint disease has been described previously. These include human papilloma virus (HPV) vaccine induced systemic lupus erythematosus and vaccines for rubella, tetanus and hepatitis B that triggered the onset and flare-up of rheumatoid arthritis [7]. Sporadic cases of giant cell arteritis and polymyalgia rheumatica occurring days after COVID-19 vaccination have also been described [8].

The pathophysiology of new onset COVID-19 vaccination related inflammatory arthritis remains to be fully elucidated. The Vaxzevria vaccine or ChAdOx1 nCoV-19 works by delivering the genetic code of the surface spike protein of SARS-CoV-2 into the recipients’ body. This spike glycoprotein then acts as an antigenic target to trigger the generation of T cells and neutralizing antibodies [4,9]. Molecular mimicry was thought to play a role in the development of autoimmunity due to the similarity of the human peptide and pathogenic element contained in the vaccine [7]. This similarity could lead to immune cross-reactivity and stimulation of an immune activation [7].

Flares of an existing autoimmune disease linked to the COVID-19 vaccine have also been reported. According to a guidance issued by the American College of Rheumatology, the possible theoretical risk of post-vaccination flare of autoimmune disease was acknowledged with moderate consensus [10]. Barbhaiya et al. identified that approximately 15% of patients with systemic rheumatic diseases attending a large rheumatology center in New York self-reported a disease flare after their COVID-19 vaccination. Disease flares occurred only after their first (23.0%) or second (4.5%) doses, or after both doses (32.7%) [11]. Flare-ups typically occur two to seven days after vaccination with moderate to severe symptoms. The majority of flare ups of existing rheumatic disease resolved within seven days of symptom onset; however, approximately 10% of patients experienced symptoms lasting longer than three weeks [11].

It is unknown whether COVID-19 vaccination might trigger or provoke flares of underlying autoimmune disease due to a non-specific adjuvant effect or immune system activation, including ANCA production [12,13]. Future research is needed to elucidate the pathogenesis and predictors of post-vaccination new-onset or flare-up of inflammatory rheumatological conditions, including to define the optimal approach to management.

## Conclusions

Flare up or new-onset Inflammatory arthritis is a rare reported side effect of COVID-19 vaccination. The majority of cases occur two to seven days after receiving the vaccine, but they can occur later. In our patient, it occurred 10 days after vaccination. In the majority of cases, symptoms resolve within a week, but they may last longer than three weeks in a small minority. 
